# Amygdala signal abnormality and cognitive impairment in drug-naïve schizophrenia

**DOI:** 10.1186/s12888-023-04728-6

**Published:** 2023-04-05

**Authors:** Huagui Guo, Haibiao Ye, Zhijian Li, Xuesong Li, Wei Huang, Yu Yang, Guojun Xie, Caixia Xu, Xiaoling Li, Wenting Liang, Huan Jing, Chunguo Zhang, Chaohua Tang, Jiaquan Liang

**Affiliations:** Department of Psychiatry, The Third People’s Hospital of Foshan, Guangdong, People’s Republic of China

**Keywords:** Schizophrenia, MRI, Cognition, Amygdala, PANSS, RBANS

## Abstract

**Background:**

Recently studies had showed that the amygdala may take part in the cognitive impairment in schizophrenia (SC). However, the mechanism is still unclear, so we explored the relationship between the amygdala resting state magnetic resonance imaging (rsMRI) signal and cognitive function, to provide a reference for the follow-up study.

**Methods:**

We collected 59 drug-naïve SCs and 46 healthy controls (HCs) from the Third People’s Hospital of Foshan. The rsMRI technique and automatic segmentation tool were used to extract the volume and functional indicators of the SC’s amygdala. The Positive and Negative Syndrome Scale (PANSS) was used to assess the severity of the disease, and the Repeatable Battery for the Assessment of Neuropsychological Status (RBANS) was used to assess cognitive function. Pearson correlation analysis was used to compare the relationship between the structural and functional indicators of the amygdala and PANSS and RBANS.

**Results:**

(1) There was no significant difference between SC and HC in age, gender and years of education. Compared with HC, the PANSS score of SC increased and the RBANS score decreased significantly. Meanwhile, the left amygdala volume decreased (t=-3.675, p < 0.001), and the Fractional amplitude of low-frequency fluctuations (FALFF) values of bilateral amygdala increased (t_L_=3.916, p < 0.001; t_R_=3.131, p = 0.002). (2) The volumes of the left amygdala were negatively correlated with the PANSS score (r_L_=-0.243, p = 0.039). While the FALFF values of the bilateral amygdala were positively correlated with the PANSS score (r_L_=0.257, p = 0.026; r_R_=0.259, p = 0.026). Bilateral amygdala volumes and FALFF values were positively correlated (r_L_=0.445, p < 0.001; r_R_=0.326, p = 0.006) and negatively correlated with RBANS score (r_L_=-0.284, p = 0.014; r_R_=-0.272, p = 0.020), respectively.

**Conclusion:**

The abnormal volume and function of the amygdala play important roles in the disease process of SC, and are closely related to cognitive impairment.

## Background

Schizophrenia (SC) is a mental disorder that makes the brain decline, involving abnormalities in multiple brain regions, including the prefrontal cortex (PFC) [1] and hippocampus [2]. The amygdala, another brain region, has attracted more and more researchers’ attention [3], because it is related to the pathophysiology of schizophrenia [4]. The amygdala was first discovered by Burdach in the 19th century. It is located in the amygdala-like brain area deep in the temporal lobe of the brain, which is now the basolateral amygdala (BLA) [5]. Subsequently, the structures around the BLA were also found [6, 7]. In these subregions, BLA, central amygdala (CeA) and medial amygdala (MeA) participate and regulate the stress response of the body when facing the stressor [8]. BLA receives sensory stimuli transmitted from sensory-related cortical regions, thus driving neuronal activity and mediating emotional behavior [3]. Abnormalities in the structure and function of the amygdala related to emotional expression disorder in SC studies [9, 10]. The results of Ho NF et al. showed that the volumes of the bilateral amygdala in SC were smaller than that in healthy people [4, 11]. However, the study of Killgore WD et al. only proved that the volume of the amygdala in SC patients could predict memory function, but there was no significant reduction in its volume [12]. At the same time, Mukherjee P et al. found that the functional connection between the amygdala and other brain regions in SC was weakened [13, 14]. Other studies suggested that when patients were angry, the activation of neural activities in the amygdala related to executive function increased [15]。.

Therefore, the abnormality of amygdala function may be the basis of SC’s emotional disharmony, social function and cognitive deficits. At present, the relationship between structural and functional abnormalities of the amygdala and cognitive impairment is still not clear. So we used rsMRI to scan the drug- naïve SC patients, and analyzed the correlation between amygdala abnormalities and cognition in SC patients, to provide reference for the follow-up study of amygdala mechanisms.

## Methods

SCs (n = 70) were from the Third People’s Hospital of Foshan (Foshan Mental Health Center). Inclusion criteria: ①conformed to the diagnostic criteria of SC in the Diagnostic and Statistical Manual of Mental Disorders 5 (DSM-5); ②age: 18–45 (in order to avoid physical diseases caused by aging); ③years of education ≥ 9 years; ④Han nationality, right-handed; ⑤no drugs for mental diseases were used before data collection. And we had to exclude 11 patients with severe behavioral disorders (destruction or violence), who could not cooperate in completing MRI scanning.

Healthy controls (HCs) (n = 46) were recruited from local communities. Age, gender and years of education correspond to the SC group, Han nationality, and right-handedness.

Exclusion criteria: contraindications of MRI scanning, brain organic and somatic diseases, history of substance (drug, alcohol) abuse, history of brain trauma, nervous system diseases, etc.

Scale assessment: The severity of the disease was assessed by the Positive and Negative Syndrome Scale (PANSS) [16]. The cognitive function of subjects was evaluated by the Repeatable Battery for the Assessment of Neuropsychological Status (RBANS) [17], which consists of 12 test tasks to assess immediate memory, visual spatial structure, language, attention and delayed memory function, and the higher scores indicate better cognition.

MRI scanning (3.0 Tesla, General Electric, United States), data processing and statistics: 3D structure MRI scanning parameters: Time repetition (TR) = 8.6 ms, Echo time (TE) = 3.3 ms, Flip angle (FA) = 12°, Field of view (FOV) = 256 mm*256mm, matrix = 256*256, layer thickness = 1 mm, layer spacing = 0 mm, slice number = 172. MRI scanning parameters of resting brain function: TR = 2000 ms, TE = 30 ms, FA = 90º, FOV = 240 mm*240 mm, matrix = 64*64, layer thickness = 4 mm, number of layers = 36, layer spacing = 1 mm, Continuous collection of 250 time point data. SPM8 (http://www.fil.ion.ucl.ac.uk/spm), cat12 (http://www.neuro.uni-jena.de/cat12), and the Data Processing Assistant for Resting-State fMRI DPARSF (http://rfmri.org/dpabi) software were used to preprocess MRI data, such as slice timing, head motion correction, spatial standardization, smoothing, filtering, and linear drift removal. Brain structure MRI data were mainly used to measure the volume of amygdala gray matter (calculated according to the Automated Anatomical Labeling (AAL) atlas [18]). The analysis and processing of brain functional MRI data included measuring the local neural activity of amygdala with the Fractional amplitude of low-frequency fluctuations (FALFF) and regional homogeneity (ReHo).

FALFF: removed the linear drift of the preprocessed data, divided the energy of each frequency in the low-frequency range (0.01 Hz < f < 0.1 Hz) by the energy of each frequency in the whole frequency range to obtain the FALFF value of each voxel, and divided it by the mean of the whole brain signal amplitude, so as to reduce the difference in the overall level of the whole brain FALFF value.

ReHo: took 27 voxels as a cluster, and used Kendall coefficient of concordance (KCC) as an indicator to measure the local consistency of each voxel and the other 26 neighboring voxels. Used the default standard brain model of the DPARSF software to obtain the KCC map of each subject. Then, divided the KCC value of the brain by the mean value in the standard brain model to achieve standardization. Finally, the mReHo maps were smoothed.

All subjects were required to complete the evaluation of the scale and the data collection of MRI within one day, and they would receive a meal and transportation subsidy of 200 yuan after completing the tests.

### Statistical analyses

Statistical Product and Service Solutions 21 (SPSS 21 (https://www.ibm.com/analytics/spss-statistics- software) was used to analyze the score of the clinical scale. The Kolmogorov-Smirnov test (K-S test) results of each measurement data of SC and HC groups showed that they all obeyed normal distribution. Two sample t-test was used to compare the structure and function of the amygdala in SC and HC by using SPM8. Chi-square test was used to compare gender. Then, Pearson correlation analysis was conducted between the volume and functional value of the amygdala and clinical data. Next, the obtained p values were then corrected by multiple comparison correction (False Discovery Rate, FDR).

## Results

**Fig. 1:**
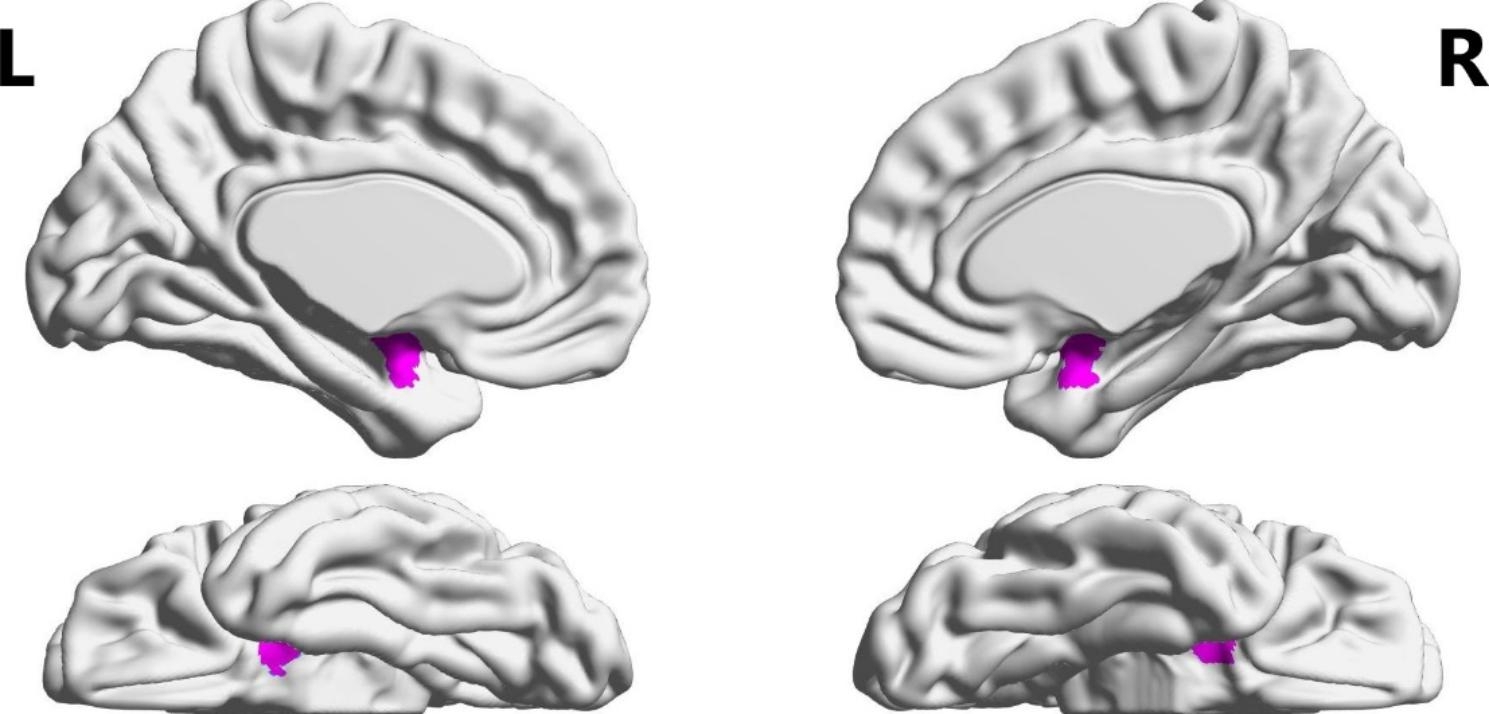
Anatomical location of the bilateral amygdala in the brain region. L: left; R: right

SC had extensive cognitive impairment, and the structural and functional impairment of the amygdala brain area was also obvious. There was no significant difference in age, gender and years of education between SC and HC. Compared with HC, PANSS score increased (t = 16.541, p < 0.001) and RBANS score decreased (t=-9.320, p < 0.001) significantly. Meanwhile, the left amygdala volume decreased (t=-3.675, p < 0.001), and the FALFF values of bilateral amygdala increased (t_L_=3.916, p < 0.001; t_R_=3.131, p = 0.002). However, there is no significant difference in the ReHo values of the bilateral amygdala. (Table 1; Fig. 1)

**Table 1:** Comparison of clinical scale and MRI data between SC and HC

		SC (n = 59)	HC (n = 46)	t / χ^2^	p
Age		42.136 ± 9.587	39.391 ± 11.275	1.347	0.181
Gender (male/female)		24/35	19/27	-0.004	0.948
Education (years)		10.153 ± 3.188	11.174 ± 3.548	-1.550	0.124
PANSS		71.069 ± 16.753	30.152 ± 0.556	16.541	< 0.001*
PANSS (positive)		15.569 ± 7.370	7.022 ± 0.147	8.345	< 0.001*
PANSS (negative)		21.966 ± 5.773	7.065 ± 0.250	16.220	< 0.001*
PANSS (general)		33.517 ± 8.225	16.044 ± 0.295	14.020	< 0.001*
RBANS		121.864 ± 35.407	190.152 ± 39.498	-9.320	< 0.001*
Immediate memory (Learning)		17.509 ± 6.673	27.630 ± 7.034	-7.531	< 0.001*
Immediate memory (Story Memory)		6.034 ± 4.768	14.413 ± 5.958	-8.030	< 0.001*
Visuospatial Construction		15.237 ± 4.554	17.761 ± 2.415	-3.402	< 0.001*
Language		11.848 ± 4.242	18.283 ± 4.344	-7.632	< 0.001*
Attention (Digit Span)		10.559 ± 2.358	14.130 ± 2.177	-7.961	< 0.001*
Attention (Coding)		28.356 ± 12.299	49.804 ± 14.149	-8.299	< 0.001*
Delayed memory (List Recall)		3.119 ± 2.547	6.609 ± 3.109	-6.323	< 0.001*
Delayed memory (List Recognition)		17.458 ± 2.843	19.544 ± 1.048	-4.728	< 0.001*
Delayed memory (Story Recall)		2.966 ± 2.936	7.522 ± 3.740	-6.994	< 0.001*
Delayed memory (Figure Recall)		8.780 ± 5.590	14.457 ± 4.708	-5.526	< 0.001*
Volume (Amygdala)	L	0.906 ± 0.096	0.982 ± 0.113	-3.675	< 0.001*
	R	1.016 ± 0.101	1.045 ± 0.106	-1.421	0.158
FALFF (Amygdala)	L	-0.083 ± 0.373	-0.335 ± 0.257	3.916	< 0.001*
	R	-0.169 ± 0.329	-0.347 ± 0.232	3.131	0.002*
ReHo (Amygdala)	L	-0.499 ± 0.339	-0.586 ± 0.291	1.395	0.166
	R	-0.537 ± 0.252	-0.559 ± 0.246	0.451	0.653

PANSS: Positive and Negative Syndrome Scale; RBANS: Repeatable Battery for the Assessment of Neuropsychological Status; FALFF: Fractional amplitude of low-frequency fluctuations; ReHo: Regional homogeneity; L: left; R: right. *indicated p < 0.05.

The cognitive impairment of SC is closely related to the abnormality of brain structure and function. Pearson correlation analysis results showed that the volumes of the left amygdala were negatively correlated with the severity of the disease (PANSS) (r_L_=-0.243, p = 0.039). And the FALFF values of the bilateral amygdala were positively correlated with the severity of the disease (r_L_=0.257, p = 0.026; r_R_=0.259, p = 0.026). In addition, the volumes of bilateral amygdala were positively correlated with the cognitive level (RBANS) (r_L_=0.445, p < 0.001; r_R_=0.326, p = 0.006) and the values of FALFF were negatively correlated (r_L_=-0.284, p = 0.014; r_R_=-0.272, p = 0.020).

The results from sub-analyses showed that the volumes of amygdala were positively correlated with immediate memory (learning: r_L_=0.407, p < 0.001; r_R_=0.286, p = 0.014; story memory: r_L_=0.384, p < 0.001; r_R_=0.259, p = 0.026), visuospatial construction (r_L_=0.265, p = 0.023; r_R_=0.279, p = 0.017), language (r_L_=0.292, p = 0.012), attention (digit span: r_R_=0.330, p = 0.006; coding: r_L_=0.424, p < 0.001; r_R_=0.337, p < 0.001), and delayed memory (list recall: r_L_=0.374, p < 0.001; r_R_=0.285, p = 0.014; story recall: r_L_=0.450, p < 0.001; r_R_=0.285, p = 0.014; figure recall: r_L_=0.378, p < 0.001; r_R_=0.317, p = 0.006). Meanwhile, the FALFF values of amygdala were negatively correlated with visuospatial construction (r_L_=-0.274, p = 0.020; r_R_=-0.327, p = 0.006), attention (digit span: r_L_=-0.263, p = 0.025; coding: r_L_=-0.325, p = 0.006; r_R_=-0.307, p = 0.006), and delayed memory (story recall: r_L_=-0.241, p = 0.039; figure recall: r_R_=-0.276, p = 0.004). (Table 2)

**Table 2:** Pearson correlation analysis of various indicators in the amygdala brain region with the severity of SC and cognitive function

		Volume (Amygdala)		FALFF (Amygdala)		ReHo (Amygdala)	
		L	R	L	R	L	R
PANSS	r	-0.243	-0.044	0.257	0.259	0.016	-0.010
	p	0.039*	0.850	0.026*	0.026*	0.914	0.931
PANSS (positive)	r	0.186	0.246	-0.008	0.070	-0.091	0.046
	p	0.286	0.137	0.953	0.820	0.719	0.896
PANSS (negative)	r	0.024	0.030	-0.245	-0.023	-0.315	-0.294
	p	0.912	0.912	0.137	0.912	0.046*	0.066
PANSS (general)	r	0.124	0.172	-0.031	0.036	-0.175	-0.064
	p	0.574	0.333	0.912	0.912	0.325	0.836
RBANS	r	0.445	0.326	-0.284	-0.272	-0.091	0.023
	p	< 0.001*	0.006*	0.014*	0.020*	0.574	0.912
Immediate memory (Learning)	r	0.407	0.286	-0.222	-0.172	-0.077	-0.020
	p	< 0.001*	0.014*	0.065	0.162	0.680	0.912
Immediate memory (Story Memory)	r	0.384	0.259	-0.212	-0.179	-0.070	0.067
	p	< 0.001*	0.026*	0.075	0.142	0.719	0.719
Visuospatial Construction	r	0.265	0.279	-0.274	-0.327	-0.048	0.059
	p	0.023*	0.017*	0.020*	0.006*	0.836	0.766
Language	r	0.292	0.138	-0.141	-0.150	-0.033	0.027
	p	0.012*	0.286	0.279	0.241	0.896	0.912
Attention (Digit Span)	r	0.330	0.221	-0.263	-0.214	-0.182	-0.029
	p	0.006*	0.065	0.025*	0.072	0.137	0.912
Attention (Coding)	r	0.424	0.337	-0.325	-0.307	-0.141	-0.021
	p	< 0.001*	< 0.001*	0.006*	0.006*	0.279	0.912
Delayed memory (List Recall)	r	0.374	0.285	-0.169	-0.185	0.014	0.068
	p	< 0.001*	0.014*	0.172	0.136	0.914	0.719
Delayed memory (List Recognition)	r	0.156	0.040	-0.101	-0.046	-0.019	0.078
	p	0.219	0.870	0.507	0.836	0.912	0.679
Delayed memory (Story Recall)	r	0.450	0.285	-0.241	-0.192	-0.065	0.033
	p	< 0.001*	0.014*	0.039*	0.119	0.719	0.896
Delayed memory (Figure Recall)	r	0.378	0.317	-0.189	-0.276	0.013	0.066
	p	< 0.001*	0.006*	0.128	0.017*	0.914	0.719

PANSS: Positive and Negative Syndrome Scale PANSS; RBANS: Repeatable Battery for the Assessment of Neuropsychological Status; FALFF: Fractional amplitude of low-frequency fluctuations; ReHo: Regional homogeneity; L: left; R: right. *indicated the p < 0.05 (FDR correction).

## Discussion

This study used rsMRI technology and automatic segmentation tools to extract the original gray matter volume and brain function indicators of SC’s amygdala and made correlation analysis with disease severity (PANSS) and cognitive function (RBANS). The results indicated that the cognitive level of SC was significantly lower than that of HC, and the volume and function of the amygdala were significantly related to cognitive function and disease severity, which provided strong support that amygdala participated in the disease process of SC.

SC is a disease with extensive brain decline [19]. It impairs the patient’s cognitive, executive, and social functions. Combined with our research results, it suggested that SC’s immediate/ delayed memory, visual function, speech function and other cognitive functions were significantly lower than healthy people. This made patients suffering from the disease have a poor prognosis and most of them did not fully recover [20]. Even those with a good prognosis, would change their lives, including but not limited to social isolation, humiliation and the possibility of finding a partner [21]. At the same time, the unemployment rate of patients with this disease is high, resulting in poor social adaptation [22].

According to the Diagnostic and Statistical Manual of Mental Disorders 5 (DSM-5) diagnostic criteria, SC is mainly characterized by delusions, hallucinations, disordered speech and behavior and other clinical symptoms [23]. Among them, hallucinations and delusions would lead to fear in SC patients, and previous studies had shown that there was a close relationship between amygdala and fear [24]. Therefore, we compared the two indicators, FALFF and ReHo, which reflect the strength and synchronization of local neural signals in the amygdala, with HC respectively. The results showed that the bilateral amygdala neural activity of SC was enhanced compared with the HC group, but there was no significant difference in the synchronization of brain regions. The results of previous studies determined that when SC was in the acute stage, under the control of positive symptoms, the amygdala received a large number of signals from the prefrontal cortex of the brain, causing the overexpression of its functions [25–27]. When the patient mainly showed negative symptoms, the local nerve activity signal of the amygdala would also increase with the negative symptoms [28], which was consistent with our results. In addition, cortical atrophy is common in SC, which has been reflected in a large number of previous studies [25–27]. Our research further supports the occurrence of amygdala atrophy in SC, which was supported by the previous results [4, 29, 30]. Previous meta-analysis results showed that the amygdala was asymmetric in MRI volume measurement of normal adults [31]. Our research suggested that not all amygdala atrophy on both sides in SC have statistical differences compared with HC. The amygdala atrophy on the right side was obvious, which was consistent with the previous studies [32, 33]. Therefore, we concluded that amygdala plays an important role in SC, and the structure and function of the amygdala would change in the process of SC disease [34].

When we did Pearson correlation analysis between amygdala volume and cognitive function and PANSS score in SC patients. We found that the volumes of bilateral amygdala had no significant difference with the PANSS score, which was not consistent with the results of previous studies [11, 35, 36]. This might be related to the subjects we selected. Generally, the course of the drug-naïve SC was relatively short, so the abnormal brain function usually occurred at the onset stage, and the structural atrophy happened at the chronic stage [25, 37]. In terms of cognitive function, previous studies on memory showed that the performance of the hippocampus was the most relevant [36, 38, 39]. Our research on the amygdala concluded that it was also involved in memory/attention/speech and other functions. The milder the cognitive impairment, the lower the degree of amygdala atrophy. On the other hand, our study also found that the value of FALFF in the amygdala was positively correlated with the severity of the disease, and negatively correlated with memory, attention and visual span, which was also confirmed in other researchers’ studies [40, 41]. What’s more, the ReHo values of amygdala had no difference between SC and HC, and had no significant correlation with RBANS scores. As the results of Xia, Qiu, et al., the abnormality of ReHo in amygdala had not been reported in large sample SC studies [42, 43].

All in all, our results demonstrated that amygdala volume and function were significantly correlated with cognitive function in patients with SC. Therefore, amygdala plays an important role in the pathology of SC, which deserves researchers’ attention. At the same time, there are some limitations in this study: the subjects were from the southern China, which does not represent all people. And we excluded the patients who were seriously ill and couldn’t cooperate to complete the test, which might skew the results of the data. Therefore, the promotion of our conclusion still needs to be cautious.

## Data Availability

The datasets generated and/or analyzed during the current study are not publicly available due to confidentiality but are available from the corresponding author on reasonable request.
